# Impact of long-term rapamycin treatment on age-related osteoarthritis in common marmoset

**DOI:** 10.1101/2024.05.14.594256

**Published:** 2024-05-17

**Authors:** Dennis M. Minton, Aditya R. Ailiani, Michael D.K. Focht, Mariana E. Kersh, Angela J. Marolf, Kelly S Santangelo, Adam B. Salmon, Adam R. Konopka

**Affiliations:** 1 Division of Geriatrics and Gerontology, Department of Medicine, University of Wisconsin-Madison, Madison, Wisconsin, USA.; 2 Department of Mechanical Science and Engineering, University of Illinois Urbana-Champaign, Champaign, IL, USA.; 3 Carle Illinois College of Medicine, University of Illinois Urbana-Champaign, Champaign, IL, USA.; 4 Beckman Institute for Advanced Science and Technology, University of Illinois Urbana-Champaign, Champaign, IL, USA.; 5 Department of Veterinary Clinical Sciences, Ohio State University, Columbus, OH, USA.; 6 Department of Microbiology, Immunology, and Pathology, College of Veterinary Medicine and Biomedical Sciences, Colorado State University, Fort Collins, CO, USA.; 7 Barshop Institute for Longevity and Aging Studies, San Antonio, TX, USA.; 9 Department of Molecular Medicine, University of Texas Health San Antonio, San Antonio, TX, USA.; 10 Geriatric Research, Education, and Clinical Center, South Texas Veterans Healthcare System, San Antonio, TX, USA.

## Abstract

**Objective:**

Pharmacologic inhibition of the mechanistic target of rapamycin (mTOR) can attenuate experimental osteoarthritis (OA) in young, male preclinical models. However, the potential of mTOR inhibition as a therapeutic mechanism for OA remains unknown. The goal of this study was to determine if mTOR-inhibition by oral rapamycin can modify OA pathology in the common marmoset, a translational model of age-associated OA.

**Methods:**

microCT and histopathologic assessments of the knee were performed on formalin-fixed hindlimbs obtained from common marmosets treated with oral rapamycin (n=24; 1mg/kg/day) or parallel control group (n=41). Rapamycin started at 9.2±3.0 years old and lasted until death (2.1±1.5 years). In a subset of marmosets, contralateral hind limbs were collected to determine mTOR signaling in several joint tissues.

**Results:**

Rapamycin decreased P-RPS6^Ser235/36^ and increased P-Akt2^Ser473^ in cartilage, meniscus, and infrapatellar fat pad, suggesting inhibition of mTORC1 but not mTORC2 signaling. Rapamycin-treated marmosets had lower lateral synovium score versus control but there was no difference in the age-related increase in microCT or cartilage OA scores. Subchondral bone thickness and thickness variability were not different with age but were lower in rapamycin-treated geriatric marmosets, which was largely driven by females. Rapamycin also tended to worsen age-related meniscus calcification in female marmosets.

**Conclusion:**

Oral rapamycin attenuated mTORC1 signaling and may have caused feedback activation of mTORC2 signaling in joint tissues. Despite modifying site-specific aspects of synovitis, rapamycin did not modify the age-associated increase in OA in geriatric marmosets. Conversely, rapamycin may have had deleterious effects on meniscus calcification and lateral tibia subchondral bone, primarily in geriatric female marmosets.

## Introduction

Increasing age is the greatest risk factor for osteoarthritis (OA). OA further increases the age-related risk of multi-morbidity. With no available disease modifying therapies for OA, the economic and societal impacts of OA are expected to rapidly inflate with population aging. A critical barrier to the development of disease modifying therapies for human OA is a lack of models that faithfully recapitulate human OA. Recently, we found that the smallest anthropoid non-human primate, the common marmoset, replicates most aspects of human joint tissue organization and develops hallmark OA pathologies in a similar age-related fashion to humans (1). Therefore, the marmoset represents an advantageous model to translate OA therapeutics from rodents to humans.

The mechanistic target of rapamycin (mTOR) is a nutrient- and growth-factor-sensitive protein kinase that exists as two distinct complexes, mTORC1 and mTORC2. Phosphorylated mTORC1/2 substrates are abundant in OA cartilage from humans, dogs, and rodents (2–5). Phosphorylation of downstream substrates of mTORC1 (5) and mTORC2 (3) can induce and exacerbate OA in mice, while cartilage specific knockout of the entire mTOR protein kinase effectively protects against surgically induced knee OA pathology in young male mice (4). These data indicate that mTOR inhibition may have therapeutic potential for OA.

Pharmacological inhibition of mTORC1 signaling by rapamycin delays several age-related pathologies and extends lifespan in multiple model systems (6). Similarly, systemic or intra-articular treatment with rapamycin can attenuate experimentally induced OA in young, male rodents (2,7). However, this is equivocal as we and others have found worsened OA pathology after rapamycin treatment (8,9). Importantly, it remains unknown whether rapamycin can modify naturally occurring OA in older male and female subjects. Therefore, to determine if mTOR-inhibition by rapamycin can impact age-related OA in a translational model of OA, we analyzed knee joints obtained from common marmosets that received long-term oral rapamycin starting near mid-life or served as controls.

## Materials and Methods

### Animal Use and Tissue Collection

Common marmosets were housed and maintained using previously published husbandry guidelines at the Barshop Institute for Longevity and Aging Research at UT Health San Antonio until natural death or compassion euthanasia. At necropsy, hind limbs were either fixed in formalin (n=64) or frozen (n=19) and shipped to University of Wisconsin-Madison (1).

### Statistical Analysis

To evaluate the effect of Rapamycin-treatment, sex, and age, multiple linear regression models were used with main effects, 2-way interactions, and 3-way interactions. If residuals were non-normally distributed as assessed by the Kolmogorov-Smirnov test, data were square-root transformed. If square-root transformation failed to normalize residuals, data were rank-transformed. Unpaired t-tests or Mann-Whitney tests were used for pairwise comparisons between treatment groups for pooled sexes, males, and females, depending on normality of data as assessed by the Kolmogorov-Smirnov test. Because marmosets are considered ‘aged’ at 8-years-old, we previously stratified into ‘adult’ (<8-years) and ‘geriatric’ (>8-years) for grouped analysis. Due to low number of ‘adult’ rapamycin-treated marmosets, we assessed treatment effects pairwise in geriatric marmosets only. P-values ≤0.05 were considered statistically significant.

Methods regarding rapamycin treatment, radiographic, histological, and molecular outcomes are described in Supplementary Methods.

## Results

### Rapamycin treatment duration and physical characteristics.

Average treatment durations for marmosets pooled and stratified by age-group and sex are shown in Table S1. On average, marmosets were treated with rapamycin for 2.1±1.5 years. Peak body mass, lean and fat mass, blood glucose, LDL cholesterol, and triglyceride levels were also measured on a subset of marmosets as shown in Table S2 and were not significantly different between control and rapamycin.

### Molecular target engagement in joint tissues.

**Figure 1:**
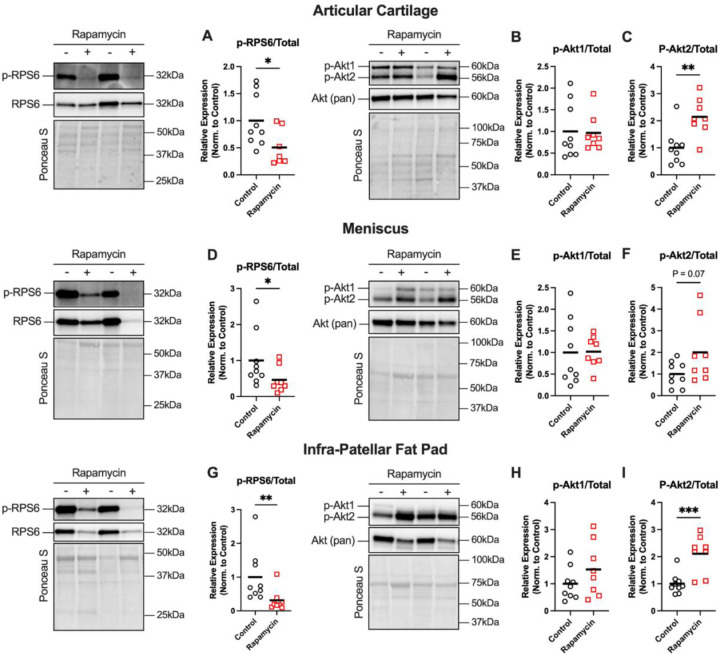
Rapamycin inhibits mTORC1 signaling in knee joint tissues. Representative western blot images from articular cartilage, meniscus, and infra-patellar fat pad tissues are shown for p-RPS6^Ser235/36^ and total RPS6, or p-Akt^Ser473^ and total Akt, with Ponceau S presented as a loading control. **A**) p-RPS6 normalized to total RPS6 was decreased in articular cartilage of Rapamycin-treated marmosets. **B**) p-Akt1 normalized to total Akt was not affected by rapa, but **C**) p-Akt2 normalized to total Akt was higher in rapamycin-treated marmosets. **D**) p-RPS6 normalized total RPS6 was decreased in meniscus of rapamycin-treated marmosets. **F**) p-Akt1 normalized to total Akt was not affected by rapa, but **H**) p-Akt2 normalized to total Akt was higher in rapamycin-treated marmosets. **G**) p-RPS6 normalized total RPS6 was decreased in infra-patellar fat pad of rapamycin-treated marmosets. **H**) p-Akt1 normalized to total Akt was not affected by rapa, but **I**) p-Akt2 normalized to total Akt was higher in rapamycin-treated marmosets. Comparisons were made using either student’s t-test Mann-Whiteny test, depending on normality of data. Data are shown as mean with individual data points. *P<0.05, **P<0.01.

Representative blots and mTOR signaling data from articular cartilage, meniscus, and infrapatellar fat pad are presented in Figure 1A-I. Rapamycin attenuated p-RPS6^Ser235/36^ (P<0.05) by ~50% versus control in cartilage, meniscus, and fat pad. Rapamycin did not affect p-Akt1^Ser473^ but increased p-Akt2^Ser473^ 2-fold versus control in cartilage (P<0.01), meniscus (P=0.07), and fat pad (P<0.01).

### Rapamycin did not modify radiographic OA score or cartilage pathology.

Total uCT OA score (Figure S1A-D) increased with age in both sexes but was not different in rapamycin-treated vs. control marmosets. Cartilage pathology (Table S3, Figure S2A-H) increased with age and manifested more severely in geriatric females but was not different in rapamycin-treated vs. control marmosets.

### Synovial pathology scores were lower in rapamycin-treated marmosets.

Total synovial score for the lateral compartment was lower in rapamycin-treated marmosets when sexes were pooled (Figure S3A, P<0.05). There were no differences between control and rapamycin-treated marmosets for the individual scoring criteria of pannus infiltration, synovial hyperplasia, or sub-synovial inflammation (Figure S3B-D); however, synovial fibrosis scores were lower in the medial compartment of rapamycin-treated males vs. male controls (Figure S3E).

### Greater meniscus calcification in rapamycin-treated geriatric marmosets.

**Figure 2:**
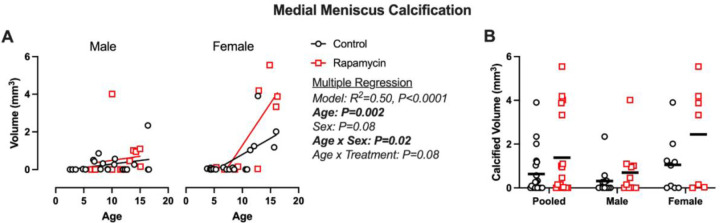
Meniscus calcification is increased in geriatric and female marmosets receiving rapamycin. **A**) Medial meniscus calcification volumes are presented as a scatter plot against age and **B**) stratified by treatment group for pooled and stratified sexes. Significant findings from multiple linear regression (A) are shown beside. Pairwise comparisons were made using Mann-Whitney tests. Data are presented as mean with individual data points.

Medial meniscus volume was predicted by multiple regression model (Figure 2A; R^2^=0.50, P<0.0001). Age (P=0.002) was a significant main effect predictor with a trend for sex (P=0.08). Interaction effects were found for age and sex (P=0.02) and a trend for sex and treatment (P=0.08), reflective of more rapamycin treated geriatric female with severe calcification. When geriatric marmosets were pooled and stratified by sex, no significant treatment effects were found (Figure 2B) despite rapamycin-treated geriatric marmosets having ~2-fold higher meniscus calcification volume than controls. No effects of age, sex, or treatment were seen in the lateral meniscus (data not shown).

### Rapamycin treated marmosets displayed altered subchondral bone architecture.

Results of multiple regression models for cortical and trabecular bone parameters are contained in Table S3 and Table S4, respectively. Lateral tibia trabecular thickness was lower in rapamycin-treated marmosets, and medial (P=0.002) and lateral (P=0.05) femoral trabecular bone mineral density was predicted by increasing age.

**Figure 3:**
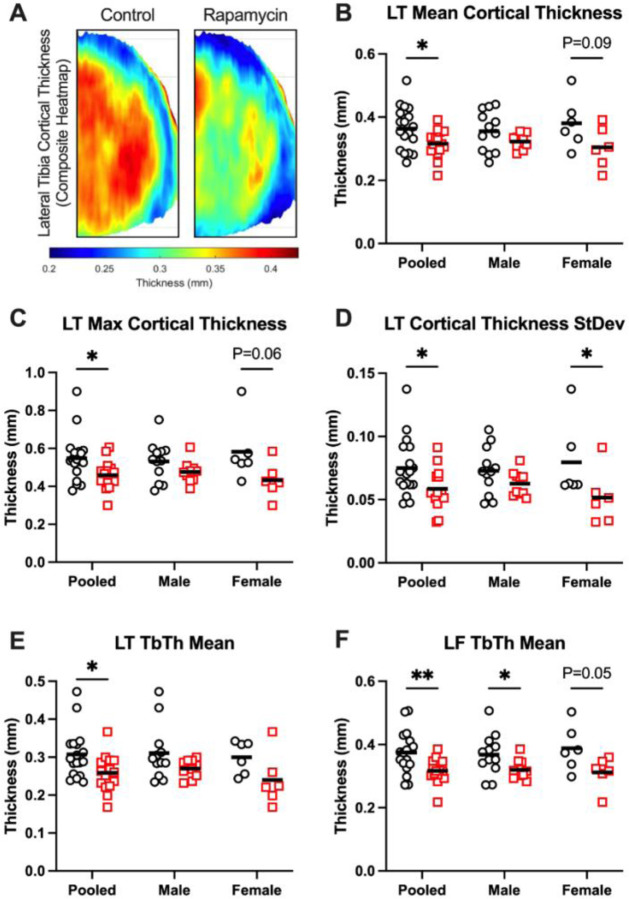
Subchondral bone architecture is altered by rapamycin treatment. **A**) Composite cortical thickness heatmaps from control and Rapamycin-treated marmosets, scalebar beneath. **B**) Mean, **C**) max, and **D**) standard deviation of lateral tibia cortical bone thickness were all lower in rapamycin-treated marmosets than controls. **E**) Trabecular thickness in the lateral tibia and **F**) lateral femur were also lower in Rapamycin-treated marmosets than controls. L=lateral, T=tibia, F=femur. Data are presented both pooled and stratified by sex. Treatment effects were assessed by unpaired t-tests or Mann-Whitney tests, depending on normality of data. Data are shown as mean with individual data points. *P<0.05, **P<0.01

When stratifying by age-groups, the lateral tibia emerged as a site with consistent rapamycin effect. Composite lateral tibia subchondral cortical thickness heatmaps for control and rapamycin-treated marmosets are shown in Figure 3A. In geriatric marmosets pooled for sex, rapamycin-treated marmosets had lower mean (Figure 3B), max (Figure 3C), and standard deviation (Figure 3D) of cortical thickness in the lateral tibia versus controls. This may have been driven by females, as rapamycin-treated females displayed lower (P=0.02) standard deviation and non-significant trends for lower mean (P=0.09) and max (P=0.06) cortical thickness.

Trabecular thickness in the lateral tibia (Figure 3E, P=0.02) and lateral femur (Figure 3F, P=0.004) were both lower in rapamycin-treated marmosets than controls when sexes were pooled. Trabecular thickness in the lateral femur was lower (P≤0.05) in rapamycin-treated males and females compared to their respective controls. No treatment effects were seen for cortical or trabecular thickness or density for any other joint compartment (data not shown).

## Discussion

The objective of this study was to examine the impact of mTOR inhibition by oral rapamycin on age-related OA pathology in common marmosets. We found that rapamycin attenuated the phosphorylation of mTORC1 substrate RPS6^Ser235/36^ and induced activation of the mTORC2 substrate Akt2^Ser473^ in multiple joint tissues. Approximately 2-years of rapamycin treatment initiated near median lifespan did not modify the age-related increase in radiographic or cartilage histopathologic OA scores but may have decreased aspects of synovitis. However, in female geriatric marmosets receiving rapamycin, medial meniscus calcification appeared to be more severe and lateral tibia subchondral cortical thickness and thickness variability were lower versus control. These effects may be maladaptive, as meniscal calcification occurs during aging and OA, and the decrease in subchondral bone thickness in the lateral tibia does not combat changes during age-associated OA. Collectively, these data indicate that mTORC1 inhibition in joint tissues by daily, oral rapamycin may lower select indices of age-related synovitis but does not offer benefits on other hallmarks of OA and may have deleterious effects on meniscus calcification and subchondral bone remodeling.

Upregulation of mTOR signaling during OA is conserved across multiple species including humans (4). Genetic deletion of the entire mTOR kinase had the strongest protective effects against surgically induced OA (4). However, the impact of pharmacological mTOR-inhibition by rapamycin on OA have been equivocal (4,5,7–9). In the present study, oral rapamycin attenuated mTORC1 and increased mTORC2 signaling as estimated by phosphorylation of downstream substrates in articular cartilage, meniscus, and infrapatellar fat pad, and did not modify radiographic or cartilage histopathologic OA scores. We have previously shown in male Dunkin-Hartley guinea pigs, dietary rapamycin (14ppm) with or without metformin (±1000ppm) decreased mTORC1 but increased mTORC2 signaling when all rapamycin-treated animals were retrospectively pooled, and this was accompanied by worsened OA pathology (9). Collectively, our data in marmosets and guinea pigs are consistent with findings that acute mTORC1 inhibition can alleviate negative feedback on upstream receptor tyrosine kinases and increase signal through mTORC2/Akt (6), which is consistent with short joint-space residence times of small drug compounds and therapeutics (1–4h) (10). Further, since constitutively activated Akt signaling can induce and worsen experimental OA in mice (3), it is plausible that increased Akt phosphorylation in rapamycin-treated marmosets may have negated any protective effects mTORC1 inhibition on age-related OA. Future work is needed to determine if inhibition of both mTORC1 and mTORC2 signaling within the joint space is needed to achieve therapeutic efficacy.

The age-related increase in medial meniscus calcification tended to be more severe in geriatric marmosets receiving rapamycin, which appeared to be driven by geriatric females. During OA, the menisci can degenerate, become calcified, and secrete inflammatory cytokines which can exacerbate OA progression. Longitudinal human data from the Multicenter Osteoarthritis Study indicate that meniscus calcification is associated with severity and progression of pain (11). While pain was not measured in the present study as originally planned due to COVID-19 restrictions, previous reports indicate an age-related increase in pain behavior linked to OA in mice and predictive of mortality in marmosets (12). The role of mTOR in meniscal calcification is not well characterized; however, mTOR inhibition by rapamycin increased calcification in bone marrow mesenchymal stem cells *in vitro* and enhanced rat mandibular trabecular bone formation during inflammatory induction with lipopolysaccharides (13). These data would support the notion that mTORC1 inhibition within a pro-inflammatory environment, as commonly seen during OA, may create a condition prone to osteogenesis and aberrant calcification.

Subchondral sclerosis is a hallmark observation during OA and is used as part of the Kellgren-Lawrence system to diagnose human OA. Rapamycin-treated marmosets had lower subchondral cortical and/or trabecular bone thickness and thickness variability in the lateral tibia and femur. While this may be viewed as resistance to sclerotic bone formation during OA, there was no age-related subchondral thickening observed at this site. Therefore, this may be a maladaptive effect of rapamycin treatment. We and others have previously observed decreased bone mass after rapamycin treatment. 12-weeks of dietary rapamycin in Dunkin-Hartley guinea pigs decreased cortical thickness but also at other non-OA-affected sites (9). Further, rapamycin (4mg/kg every other day) decreased cortical and trabecular bone thickness and increased serum CTX-1 and other indices of bone resorption in 12–16-week-old female mice (14). Further, rapamycin (5mg/kg, intra-articular, daily) caused subchondral bone loss male Sprague-Dawley rats during a TMJ-OA model (8). Together, these data support rapamycin can decrease bone mass, though this may be dependent on age, disease status, and frequency of dosing.

Some characteristics of synovitis appear to be positively impacted by rapamycin. In the lateral joint compartment, total synovial scores and fibrosis scores were lower in rapamycin-treated marmosets when sexes were pooled and in males only, respectively. While these effects were not found in the medial compartment where OA manifests most severely, there is precedent that mTOR signaling contributes to synovitis. Macrophage infiltration and polarization via hyperactive mTORC1 signaling have been shown to promote synovitis and OA progression (15), and rapamycin treatment decreased synovitis in male mice following DMM (7).

While this is the first study to evaluate the effects of rapamycin on OA outcomes in non-human primates, we acknowledge some study limitations. As demonstrated in our previous study (1), age-related OA in marmosets manifests with a heterogenous onset and progression compared to post-traumatic OA models where there is a robust and controlled stimulus to induce OA. While this is common to primary OA in mice and humans, many of the marmosets likely had some amount of age and/or OA-related changes to joint tissues when the rapamycin treatment began. Because of the cross-sectional study design, we cannot account for existing OA burden at treatment start. Therefore, these findings may not be reflective of the potential preventative effects of mTOR inhibition on OA outcomes in subjects at high risk for developing OA (i.e., ACL rupture). Future work may seek to account for existing OA burden prior to treatment through in vivo imaging and/or OA biomarkers.

Due to the known kinetics of rapamycin on mTOR signaling in other cells and tissue types, our data align with the concept that systemic rapamycin treatment may have low entry into and/or short residence time within the joint space to acutely inhibit mTORC1 and stimulate mTORC2 signaling. Therefore, alternative strategies to extend rapamycin joint residence time should be investigated to determine if inhibiting both mTORC1 and mTORC2 specifically within the joint space may increase therapeutic efficacy and minimize risk for off-target systemic metabolic side-effects. Collectively, our findings demonstrate the effects of oral rapamycin may lower select characteristics of synovitis but may also contribute to deleterious effects on meniscus calcification and subchondral bone structure.

## Supplementary Material

Supplement 1
