# Supplementary material for: Impact of long-term rapamycin treatment on age-related osteoarthritis in common marmoset
Source: bioRxiv. 2024 May 17:2024.05.14.594256. Preprint. [Version 1] doi: 10.1101/2024.05.14.594256 (PMC11118493; doi:10.1101/2024.05.14.594256)
Supplement: Supplementary file 1 [file media-1.pdf]

SUPPLEMENTARY METHODS

Rapamycin Treatment

Twenty-three (14M/9F) marmosets received eudragit-encapsulated rapamycin (1mg/kg/day) dissolved in yogurt. The control group consisted of 28 marmosets (14M/14F) that received yogurt vehicle with empty eudragit as previously published (1) and an additional 13 marmosets (7M/6F) from the general population that did not receive eudragit vehicle. All husbandry was identical and there were no differences in OA between the vehicle control and age-matched general population marmosets. Marmosets in the control group for this study were previously published to characterize OA pathology in the common marmoset (2). All samples were collected opportunistically at euthanasia determined by animals reaching veterinary end of life criteria.

Protein and RNA Extraction from Frozen Tissues

Frozen knee joints were thawed on ice for ~45-minutes and articular cartilage, menisci, and the infrapatellar fat pad were dissected and snap frozen until further processing. Tissues were placed in tubes containing 500uL Trizol (BRAND) and 500mg of 2.4mm ceramic beads and pulverized in an Omni Bead Ruptor (two 30-second cycles at 6m/s). RNA and protein were then extracted as previously described (3). RNA quantity and purity were evaluated via Nanodrop (Thermo), and protein concentration was quantified via bicinchoninic acid assay.

Western Blotting

Samples were prepared for western blotting as previously described (4). Equal amounts of protein were resolved on 4-15% TGX precast gels (BioRad) and transferred to nitrocellulose membranes (BioRad) and stained with Ponceau S to quantify total protein per lane. Membranes were blocked with 5% bovine serum albumin then incubated overnight at 4C with primary antibodies raised against p-RPS6 Ser235/236 (#4858), RPS6 (#2217), p-Akt Ser473 (#4060), and Akt (pan) (#4685) followed by an HRP-conjugated anti-Rabbit secondary antibody (#7074) for 45-min at room-temperature. All antibodies were from Cell Signaling (Danvers, MA, USA). Membranes were imaged using a BioRad Chemi-Doc after 5-min incubation in SuperSignal Femto chemiluminescent substrate (Thermo). Bands were quantified using densitometric analysis in ImageLab (BioRad).

All radiographic and histological analyses were performed as outlined in our previous manuscript (2), and all control and rapamycin-treated marmosets were processed and analyzed in parallel. Detailed methods can be found in our previous manuscript, however are replicated below for ease of readership:

microCT Acquisition and Quantitative Analysis

Knee joints were scanned while submerged in 70% ethanol using a Rigaku CT Lab GX130 at 120uA and 110kV for 14-minutes, achieving an isometric voxel size of 49um as previously performed (4). Three hydroxyapatite phantoms were included on each day of scanning to calculate bone mineral density.

For quantitative analysis, all scans were binarized using identical thresholds to allow for comparison across scans. In Amira (v2021.2, ThermoFisher), regions of calcified meniscus and were segmented, and volumes were quantified using the material statistics function. Representative 3D reconstructions were also created in Amira.

For analysis of subchondral bone, cortical and trabecular bone were separated using a previously validated algorithm (5). Three-dimensional thickness and density maps were created the ImageJ plug-in BoneJ. Articular surfaces were spatially normalized to account for morphological differences between marmosets, resulting in 81 subregions in the tibial plateaus and 40 subregions in the femoral condyles. For each sub-region, mean trabecular thickness and density were calculated. Subregions were then compiled to produce mean and max values for each joint compartment, as well as standard deviation as an index of heterogeneity of thickness and density across the articular surface. Three scans from the geriatric group could not be analyzed due to severe subchondral bone degeneration.

Semi-Quantitative microCT Scoring

To assess radiographic severity of OA, uCT scans were graded by a veterinary radiologist according to the criteria outlined in **Table S6** (6). Grading was performed in a single session utilizing standard transverse plane images and multiplanar reconstructions in sagittal and coronal planes while remaining blinded to marmoset age or sex. We also employed a semi-quantitative scoring system to evaluate calcification of the menisci. Briefly, the medial and lateral menisci were each assigned a score ranging from 0-5 depending on the size and frequency of ectopic calcification.

Histological Analysis

Following microCT scanning, knee joints were grossed of soft tissue, trimmed, and decalcified in 5% EDTA changed 2-3rx/week for 15-weeks or until fully decalcified by mechanical endpoint testing. Joints were then transected along the medial collateral ligament and processed for paraffin embedding. Sections were taken at 5-microns and stained with Toluidine Blue as previously described (2,4). In a blinded fashion, histological assessment of OA was performed using a grading scheme shown in **Table S7** which was adapted from the Modified Mankin guidelines for mice and other small rodents (7). Briefly, 3 sections across each joint compartment were graded in a blinded fashion for articular cartilage structural damage based on lesion depth and affected proportion of cartilage surface (0-11), tidemark duplication (0-3), proteoglycan loss as evident by loss of toluidine blue staining (0-8), and disruption of normal chondrocyte cellularity (0-4). Data were then presented as an average of the 3 scores. These criteria were assessed as individual phenomena and summed to produce a total histological score for each joint compartment and a whole-joint score.

Synovium Scoring

To assess OA-associated changes to the synovium/joint capsule, we employed a recently published scoring system developed to comprehensively evaluate histopathological changes in mouse models of OA (8). Scoring criteria are presented in **Table S8** but briefly included scores for pannus outgrowth and subsequent bone erosion, synovial exudate, synovial hyperplasia, sub-synovial inflammation, and scores for synovial fibrosis severity and coverage of fibrosis. Two mid-

joint coronal sections were stained with Hematoxylin & Eosin and used to produce a single score for the medial and lateral compartments for each marmoset. Synovial fibrosis severity and coverage were multiplied to produce the individual fibrosis score presented. To produce the total synovium score per compartment, all individual criteria were summed besides synovial fibrosis coverage to avoid over-representing fibrosis in the total score. Of note, some criteria are applied relative to cartilage thickness. We found marmoset cartilage to be substantially thicker than mouse, which may have decreased our ability to capture more subtle changes. However, the scoring system was still applied faithfully. Finally, because synovial exudate receives a binary score, it was not analyzed as an individual criteria but was included in the total score.

Supplementary Tables and Figures

	Male		Female		Pooled Sexes	
	N	Duration (yrs)	N	Duration (yrs)	N	Duration (yrs)
Adult	3	0.6 ± 0.3	2	1.3 ± 0.3	5	0.9 ± 0.5
Geriatric	11	2.7 ± 1.6	8	2.2 ± 1.6	19	2.5 ± 1.6
Pooled Ages	14	2.2 ± 1.7	10	2.0 ± 1.4	24	2.1 ± 1.5

Table S1. Duration of rapamycin treatment in years for marmosets presented as pooled and stratified by age-group and sex. Number of marmosets is shown for each sub-group. Data are presented as mean ± SD.

Variable	Control	Rapamycin	P-value
Peak bodyweight (g)	446.6 ± 60.5	430.1 ± 54.2	0.36
Lean mass (g)	361.1 ± 46.2	351.8 ± 41.3	0.51
Fat mass (g)	32.0 ± 31.2	33.5 ± 20.9	0.82
Blood glucose (mg/dL)	161.3 ± 53.2	199 ± 81.2	0.15
LDL cholesterol (mg/dL)	161.0 ± 42.2	188.4 ± 49.6	0.14
Triglycerides (mg/dL)	187.6 ± 149.6	281.2 ± 169	0.14

Table S2: Peak bodyweight, lean mass, fat mass, blood glucose, LDL cholesterol, and triglycerides are shown for control and rapamycin-treated marmosets. Data are presented at mean ± SD. Treatment groups were compared using 2-tailed, unpaired t-tests and P-values are presented.

Variable		Model Fit		Main Effects			2-way Interactions			3-way Interactions
		R^2	P	Age	Sex	Treatment	Age x Sex	Age x Treatment	Sex x Treatment	Age x Sex x Treatment
Medial Tibia	CoTh Mean	0.06	0.86	P=0.46	P=0.96	P=0.60	P=0.77	P=0.69	P=0.50	P=0.63
	CoTh Max	0.18	0.15	P=0.09	P=0.56	P=0.10	P=0.48	P=0.12	P=0.59	P=0.68
	CoTh StDev	0.17	0.19	P=0.12	P=0.57	P=0.08	P=0.54	P=0.09	P=0.35	P=0.44
	CoBMD Mean	0.08	0.72	P=0.87	P=0.81	P=0.95	P=0.90	P=0.67	P=0.88	P=0.77
	CoBMD Max	0.06	0.85	P=0.90	P=0.86	P=0.96	P=0.98	P=0.83	P=0.96	P=0.96
	CoBMD StDev	0.16	0.24	P=0.08	P=0.38	P=0.41	P=0.23	P=0.25	P=0.59	P=0.70
Medial Femur	CoTh Mean	0.05	0.90	P=0.61	P=0.87	P=0.95	P=0.96	P>0.99	P=0.50	P=0.55
	CoTh Max	0.08	0.70	P=0.83	P=0.54	P=0.45	P=0.74	P=0.49	P=0.64	P=0.64
	CoTh StDev	0.07	0.79	P=0.45	P=0.22	P=0.57	P=0.31	P=0.61	P=0.79	P=0.85
	CoBMD Mean	0.06	0.85	P=0.56	P=0.37	P=0.96	P=0.78	P=0.88	P=0.99	P=0.95
	CoBMD Max	0.04	0.93	P=0.78	P=0.81	P=0.43	P=0.84	P=0.59	P=0.82	P>0.99
	CoBMD StDev	0.12	0.42	P=0.07	P=0.52	P=0.07	P=0.47	P=0.10	P=0.51	P=0.58
Lateral Tibia	CoTh Mean	0.09	0.66	P=0.55	P=0.57	P=0.64	P=0.75	P=0.86	P=0.72	P=0.57
	CoTh Max	0.18	0.14	P=0.67	P=0.54	P=0.61	P=0.78	P=0.41	P=0.62	P=0.46
	CoTh StDev	**0.30**	**0.008**	P=0.25	P=0.60	P=0.42	P=0.71	P=0.86	P=0.20	P=0.18
	CoBMD Mean	0.12	0.44	P=0.40	P=0.14	P=0.75	P=0.53	P=0.97	P=0.71	P=0.58
	CoBMD Max	0.10	0.58	P=0.48	P=0.36	P=0.98	P=0.68	P=0.79	P=0.75	P=0.63
	CoBMD StDev	0.19	0.12	P=0.05	P=0.57	P=0.01	P=0.99	P=0.02	P=0.08	P=0.17
Lateral Femur	CoTh Mean	0.07	0.81	P=0.62	P=0.51	P=0.33	P=0.70	P=0.46	P=0.86	P=0.81
	CoTh Max	0.17	0.18	P=0.23	P=0.52	P=0.32	P=0.98	P=0.34	P=0.97	P=0.97
	CoTh StDev	0.17	0.20	P=0.53	P=0.66	P=0.33	P=0.26	P=0.33	P=0.58	P=0.46
	CoBMD Mean	0.15	0.29	P=0.26	P=0.05	P=0.43	P=0.18	P=0.36	P=0.50	P=0.54
	CoBMD Max	0.06	0.81	P=0.61	P=0.56	P=0.27	P=0.88	P=0.32	P=0.45	P=0.39
	CoBMD StDev	0.09	0.63	P=0.94	P=0.83	P=0.15	P=0.77	P=0.19	P=0.32	P=0.34

Table S3. Results from multiple linear regression model with model fit, main effects, and interaction effects between age, sex, and rapamycin treatment are shown for subchondral cortical bone analyses. Co=cortical, Th=Thickness, BMD=bone mineral density. Relationships where P≤0.05 are highlighted in **red**, and trends where P≤0.1 but >0.05 are **bolded**. Individual main effect and interaction predictors are similarly highlighted upon significant model fit.

Variable		*Model Fit*		*Main Effects*			*2-way Interactions*			*3-way Interactions*
		R^2	P	Age	Sex	Treatment	Age x Sex	Age x Treatment	Sex x Treatment	Age x Sex x Treatment
MT	TbTh Mean	**0.21**	**0.08**	P=0.13	P=0.95	P=0.15	P=0.82	P=0.29	P=0.07	P=0.15
	Tb BVF	**0.11**	**0.08**	P=0.32	P=0.90	P=0.59	P=0.83	P=0.53	P=0.74	P=0.73
	TbBMD	**0.22**	**0.07**	**P=0.04**	P=0.67	P=0.27	P=0.86	P=0.45	P=0.25	P=0.60
MF	TbTh Mean	0.10	0.61	P=0.58	P=0.51	P=0.42	P=0.70	P=0.61	P=0.71	P=0.95
	Tb BVF	0.17	0.18	P=0.42	P=0.77	P=0.23	P=0.98	P=0.22	P=0.86	P=0.92
	Tb BMD	**0.34**	**0.002**	**P=0.002**	P=0.33	P=0.61	P=0.95	P=0.59	P=0.87	P=0.62
LT	TbTh Mean	**0.32**	**0.003**	P=0.39	P=0.67	**P=0.02**	P=0.73	**P=0.10**	P=0.15	P=0.10
	Tb BVF	0.11	0.54	P=0.99	P=0.36	P=0.14	P=0.38	P=0.17	P=0.28	P=0.28
	Tb BMD	0.13	0.38	P=0.26	P=0.88	P=0.92	P=0.60	P=0.67	P=0.81	P=0.38
LF	TbTh Mean	0.17	0.20	P=0.67	P=0.29	P=0.28	P=0.22	P=0.57	P=0.39	P=0.30
	Tb BVF	0.08	0.69	P=0.47	P=0.41	P=0.86	P=0.72	P=0.80	P=0.94	P=0.66
	Tb BMD	**0.29**	**0.01**	**P=0.05**	P=0.87	P=0.27	P=0.30	P=0.27	P=0.32	P=0.16

Table S4. Results from multiple linear regression model with model fit, main effects, and interaction effects between age, sex, and rapamycin treatment are shown for subchondral trabecular bone analyses. M=medial, L=lateral, T=tibia, F=femur, Tb=trabecular, Th=Thickness, BVF=bone volume fraction, BMD=bone mineral density. Relationships where P≤0.05 are highlighted in **red**, and trends where P≤0.1 but >0.05 are **bolded**. Individual main effect and interaction predictors are similarly highlighted upon significant model fit.

Variable		*Model Fit*		*Main Effects*			*2-way Interactions*			*3-way Interactions*
		R^2	P	Age	Sex	Treatment	Age x Sex	Age x Treatment	Sex x Treatment	Age x Sex x Treatment
WJ	Total	**0.24**	**0.02**	**P=0.002**	P=0.19	P=0.86	**P=0.04**	P=0.78	P=0.68	P=0.42
	ACS	**0.25**	**0.02**	**P=0.0009**	P=0.11	P=0.71	**P=0.03**	P=0.54	P=0.41	P=0.32
	PG	**0.20**	**0.07**	**P=0.006**	P=0.16	P=0.69	**P=0.05**	P=0.97	P=0.68	P=0.46
	CC	0.18	0.13	P=0.02	P=0.48	P=0.85	P=0.29	P=0.93	P=0.81	P=0.71
MT	Total	**0.30**	**0.004**	**P=0.002**	P=0.16	P=0.48	**P=0.06**	P=0.64	P=0.66	P=0.52
	ACS	**0.28**	**0.008**	**P=0.004**	P=0.24	P=0.51	P=0.12	P=0.63	P=0.77	P=0.67
	PG	**0.27**	**0.009**	**P=0.004**	P=0.25	P=0.64	**P=0.08**	P=0.80	P=0.50	P=0.36
	CC	**0.24**	**0.02**	**P=0.005**	P=0.25	P=0.64	**P=0.08**	P=0.80	P=0.50	P=0.36
MF	Total	**0.21**	**0.06**	**P=0.003**	P=0.22	P=0.80	**P=0.05**	P=0.81	P=0.58	P=0.95
	ACS	**0.29**	**0.004**	**P=0.003**	P=0.60	P=0.65	P=0.43	P=0.92	P=0.19	P=0.38
	PG	0.10	0.49	P=0.03	P=0.23	P=0.80	P=0.16	P=0.67	P>0.99	P=0.89
	CC	0.11	0.42	P=0.05	P=0.47	P=0.81	P=0.29	P=0.98	P=0.76	P=0.94
LT	Total	0.10	0.49	P=0.05	P=0.32	P=0.58	P=0.18	P=0.53	P=0.43	P=0.45
	ACS	0.19	0.10	P=0.01	P=0.08	P=0.19	P=0.07	P=0.28	P=0.11	P=0.21
	PG	0.12	0.37	P=0.04	P=0.22	P=0.78	P=0.10	P=0.74	P=0.66	P=0.47
	CC	0.06	0.85	P=0.61	P=0.82	P=0.70	P=0.84	P=0.65	P=0.96	P=0.86
LF	Total	**0.22**	**0.05**	**P=0.03**	P=0.62	P=0.16	P=0.14	P=0.59	P=0.66	P=0.87
	ACS	**0.24**	**0.02**	**P=0.03**	P=0.67	P=0.19	P=0.35	P=0.24	P=0.56	P=0.49
	PG	0.16	0.18	P=0.03	P=0.48	P=0.45	P=0.18	P>0.99	P=0.97	P=0.65
	CC	0.18	0.11	P=0.005	P=0.18	P=0.83	P=0.08	P=0.34	P=0.37	P=0.21

Table S5. Results from multiple linear regression model with model fit, main effects, and interaction effects between age, sex, and rapamycin treatment are shown for histological analyses for the whole-joint and individual compartments. WJ-whole joint, M=medial, L=lateral, T=tibia, F=femur. Relationships where P≤0.05 are highlighted in **red**, and trends where P≤0.1 but >0.05 are **bolded**. Individual main effect and interaction predictors are similarly highlighted upon significant model fit.

Table S6: microCT scoring system for radiographic evaluation of OA.

**MicroCT Finding**	**Score**
Osteophyte location	1 = medial/lateral tibia<br>2 = patella<br>3 = medial/lateral femur
Osteophyte size	0 = none<br>1 = small (<1mm)<br>3 = large (≥1mm)
Subchondral bone cystic changes	0 = no<br>1 = yes
Subchondral bone sclerosis	0 = no<br>1 = yes
Articular bone lysis	0 = no<br>1 = yes
Intra-articular soft tissue	0 = normal<br>1 = increased

Table S7: Grading criteria for histological evaluation of OA in marmosets.

Parameter	Grade	Description
Articular Cartilage Structure	0	Normal
	1	Undulating articular surface, but no fibrillation
	2	Mild superficial fibrillation involving < half of the plateau/condyle
	3	Mild superficial fibrillation involving > half of the plateau/condyle
	4	Mild fibrillation/clefts/loss involving up to 1/3 depth of articular cartilage thickness in < half of plateau/condyle
	5	Mild fibrillation/clefts/loss involving up to 1/3 depth of articular cartilage thickness in ≥ half of plateau/condyle
	6	Moderate fibrillation/clefts/loss involving up to 2/3 depth of articular cartilage thickness in < half of the plateau/condyle
	7	Moderate fibrillation/clefts/loss involving up to 2/3 depth of noncalcified articular cartilage thickness in & half of the plateau/condyle
	8	Severe fibrillation/clefts/loss involving > 2/3 depth of articular cartilage thickness in < half of plateau/condyle
	9	Severe fibrillation/clefts/loss involving > 2/3 depth of articular cartilage thickness in > half of plateau/condyle
	10	Clefts/loss of articular cartilage through tidemark
	11	Clefts/loss of articular cartilage through to subchondral bone
Tidemark Duplication	0	None (only one tidemark)
	1	2 tidemarks
	2	>2 tidemarks
	3	No visible tidemark remaining
Toluidine Blue Staining	0	Normal (no loss of staining in articular cartilage)
	1	Moderate loss of staining in up to 1/2 depth of articular cartilage thickness and involving < half of the plateau/condyle
	2	Moderate loss of staining in up to 1/2 depth of articular cartilage thickness involving > half of the plateau/condyle
	3	Moderate loss of staining in > 1/2 depth of articular cartilage thickness and involving < half of plateau/condyle

	4	Moderate loss of staining in > 1/2 depth of articular cartilage thickness and involving 2 half of plateau/condyle
	5	Severe loss of staining up to 1/2 depth of articular cartilage thickness and involving < half of plateau/condyle
	6	Severe loss of staining up to 1/2 depth of articular cartilage thickness and involving ≥ half of plateau/condyle
	7	Severe loss of staining in >1/2 depth of articular cartilage thickness and involving < half of the plateau/condyle
	8	Severe loss of staining in > 1/2 depth of articular cartilage thickness and involving ≥ half of plateau/condyle
Chondrocyte Cellularity	0	0-5 groupings of hypertrophic chondrocytes or chondrones containing <3 cells.
	1	6-10 groupings in chondrones or clusters containing 3 or more cells.
	2	>10 groupings of chondrones or clusters containing 3 or more cells.
	3	Loss of cellularity in <1/2 of the plateau or condyle.
	4	Loss of cellularity in 1/2 or greater of the plateau or condyle.

Table S8: Grading criteria for evaluation of synovium/joint capsule.

Parameter	Grade	Description
Pannus Infiltration	0	No pannus
	1	Pannus >2 cells thickness is present on the bone at the joint margin but has not migrated over the cartilage surface.
	2	Pannus has migrated over the cartilage surface OR invaded into the cartilage < 1x cartilage depth.
	3	Pannus has migrated over the cartilage surface OR invaded into the cartilage > 1x cartilage depth.
Bone Erosion	0	No cortical bone erosion at any site.
	1	Partial thickness loss of cortical bone only.
	2	Focal complete loss of cortical bone. Communication with the marrow cavity at only one small vascular communication site.
	3	Widespread complete loss of cortical bone. Multiple sites of communication with marrow cavity or broad area loss of cortical bone.
Synovial Hyperplasia	0	1 cell thick
	1	2-3 cells thick
	2	4-5 cells thick
	3	>5 cells thick
Sub-Synovial Inflammation	0	No inflammatory cells
	1	Occasional scattered inflammatory cells – perivascular or scattered throughout.
	2	Focal areas of dense sub-synovial white blood cell infiltrate but still predominantly normal sub-synovial areolar connective tissue present.
	3	Widespread dense sub-synovial white blood cell infiltrate. Markedly reduced or little/no normal areolar connective tissue OR lymphoid follicle formation – distinct accumulation of mononuclear cells organized into rounded masses reminiscent of lymph nodules.
Synovial Exudate	0	No inflammatory cells or fibrin in the synovial cavity.
	1	Inflammatory cells and/or fibrin clot in the synovial cavity.
Synovial/Joint Capsule Fibrosis (Severity)	0	< 1/3x cartilage thickness
	1	1/3-2/3x cartilage thickness but not densely packed fibrous tissue.
	2	1/3-2/3x cartilage thickness and densely packed fibrous tissue.
	3	2/3-1x cartilage thickness but not densely packed fibrous tissue.
	4	2/3-1x cartilage thickness and densely packed fibrous tissue.
	5	>1x cartilage thickness but not densely packed fibrous tissue.
	6	>1x cartilage thickness and densely packed fibrous tissue.

Synovial/Joint Capsule Fibrosis (Coverage)	0	<20%
	1	21-40%
	2	41-60%
	3	61-80%
	4	>80%

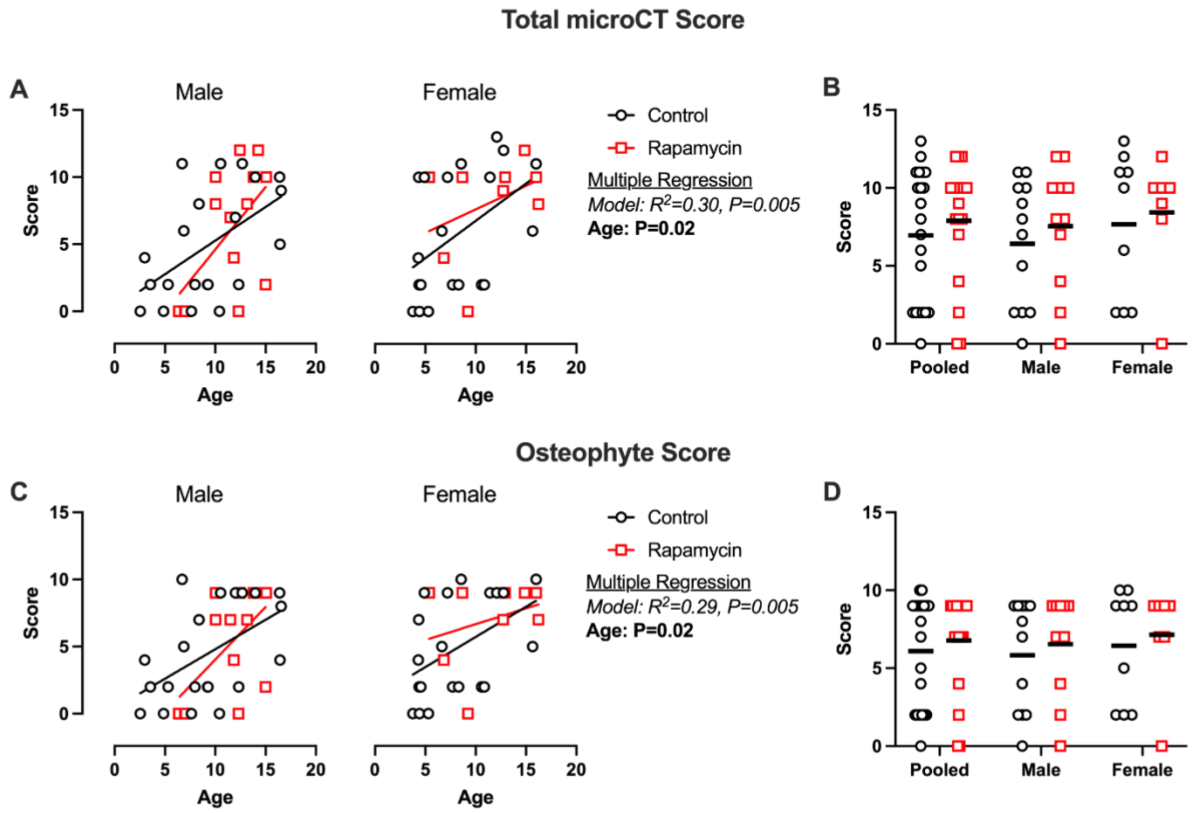

Figure S1: Radiographic OA is similar between control and rapamycin-treated marmosets. **A**) Total uCT OA scores presented as scatter plots against age and **B**) stratified by treatment group for geriatric males, females, and pooled sexes. **C**) Osteophyte scores are similarly presented as scatter plots against age and **D**) stratified by treatment group for geriatric males, females, and pooled sexes. Results of multiple linear regression (A,C) are shown beside graphs. Comparisons between groups were made using Mann-Whitney tests (B,D). Data are presented as mean with individual data points.

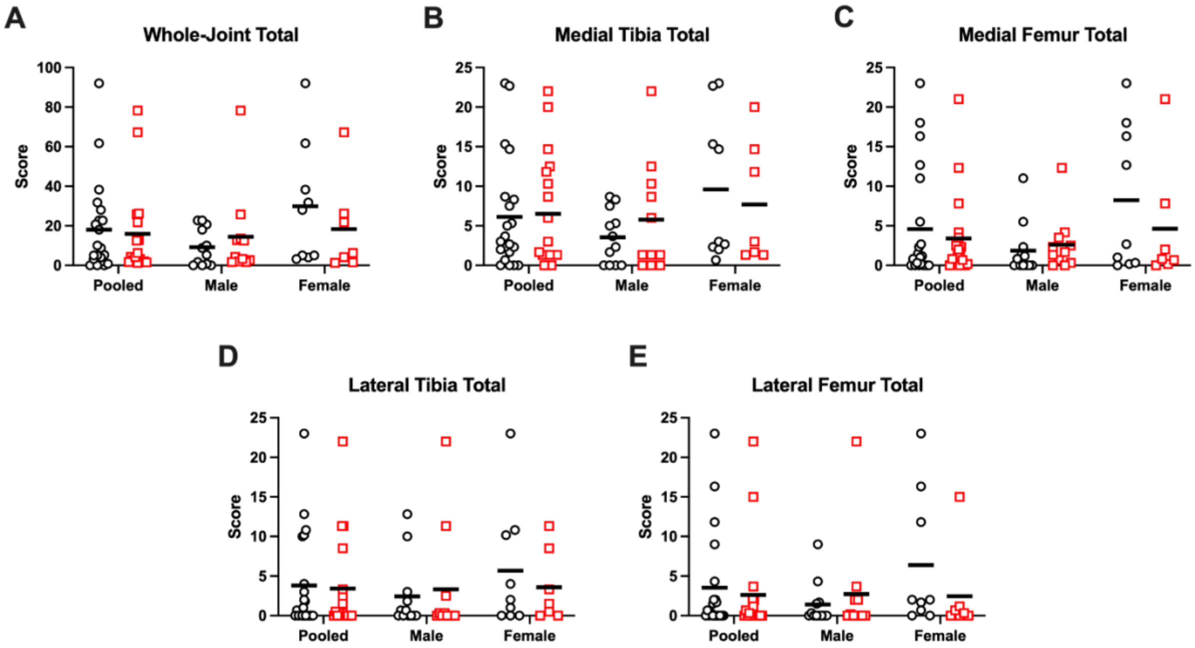

Figure S2: Cartilage histopathology was not affected by rapamycin. **A**) Total histological scores for the whole-joint, **B**) medial tibia and **C**) femur, and **D**) lateral tibia and **E**) femur are compared between control and rapamycin-treated geriatric marmosets when pooled and stratified by sex. Controls are presented as Comparisons were made using Mann-Whitney tests. Data are presented as mean with individual data points.

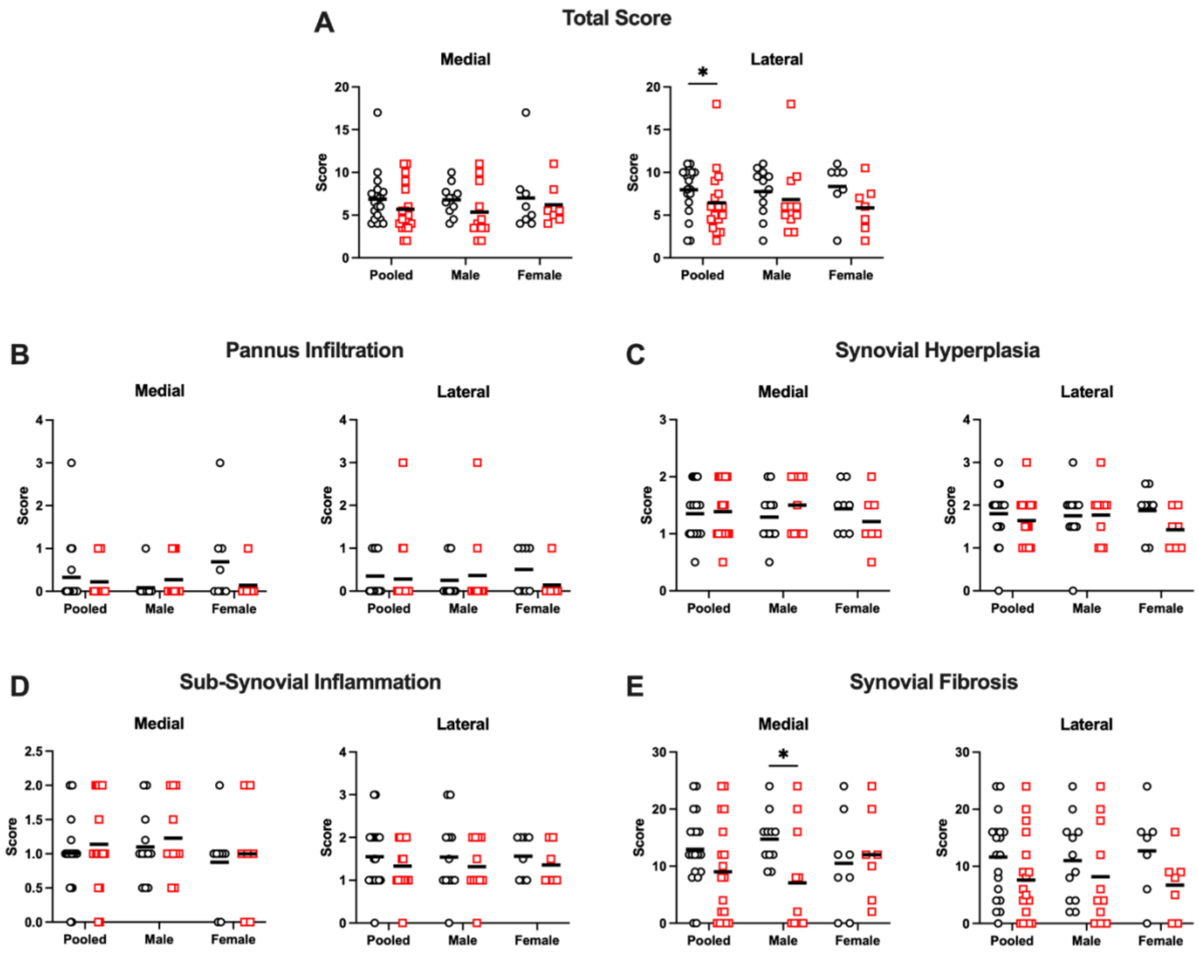

Figure S3: Effect of rapamycin on synovitis. **A**) Total synovial score and the individual scoring criteria of **B**) pannus infiltration, **C**) synovial hyperplasia, **D**) sub-synovial inflammation, and **E**) synovial fibrosis for the medial and lateral compartments are shown for geriatric control and rapamycin-treated marmosets pooled and stratified by sex. Comparisons were made using unpaired t-tests or Mann-Whitney tests, depending on normality of data. Data are presented as mean with individual data points. *P<0.05

References

1. Tardif S, Ross C, Bergman P, Fernandez E, Javors M, Salmon A, et al. Testing efficacy of administration of the antiaging drug rapamycin in a nonhuman primate, the common marmoset. J Gerontol A Biol Sci Med Sci. 2015 May;70(5):577–87.

2. Minton DM, Ailiani AR, Focht MDK, Kersh ME, Marolf AJ, Santangelo KS, et al. The common marmoset as a translational model of age-related osteoarthritis. GeroScience [Internet]. 2024 Mar 11 [cited 2024 Mar 20]; Available from: https://doi.org/10.1007/s11357-024-01103-5

3. Kobak KA, Batushansky A, Borowik AK, Lopes EPB, Peelor III FF, Donovan EL, et al. An In Vivo Stable Isotope Labeling Method to Investigate Individual Matrix Protein Synthesis, Ribosomal Biogenesis, and Cellular Proliferation in Murine Articular Cartilage. Function. 2022 Mar 23;3(2):zqac008.

4. Minton DM, Elliehausen CJ, Javors MA, Santangelo KS, Konopka AR. Rapamycin-induced hyperglycemia is associated with exacerbated age-related osteoarthritis. Arthritis Res Ther. 2021 Dec;23(1):253.

5. Ang IC, Fox M, Polk JD, Kersh ME. An Algorithm for Automated Separation of Trabecular Bone From Variably Thick Cortices in High-Resolution Computed Tomography Data. IEEE Transactions on Biomedical Engineering. 2020 Mar;67(3):924–30.

6. Radakovich LB, Marolf AJ, Shannon JP, Pannone SC, Sherk VD, Santangelo KS. Development of a microcomputed tomography scoring system to characterize disease progression in the Hartley guinea pig model of spontaneous osteoarthritis. Connective Tissue Research. 2018 Nov 2;59(6):523–33.

7. Furman BD, Strand J, Hembree WC, Ward BD, Guilak F, Olson SA. Joint degeneration following closed intraarticular fracture in the mouse knee: A model of posttraumatic arthritis. J Orthop Res. 2007 May;25(5):578–92.

8. Haubruck P, Heller R, Blaker CL, Clarke EC, Smith SM, Burkhardt D, et al. Streamlining quantitative joint-wide medial femoro-tibial histopathological scoring of mouse post-traumatic knee osteoarthritis models. Osteoarthritis and Cartilage. 2023 Dec 1;31(12):1602–11.
